# The Diagnostic Accuracy of Mutant KRAS Detection from Pancreatic Secretions for the Diagnosis of Pancreatic Cancer: A Meta-Analysis

**DOI:** 10.3390/cancers12092353

**Published:** 2020-08-20

**Authors:** Nikhil Patel, Tatjana Petrinic, Michael Silva, Zahir Soonawalla, Srikanth Reddy, Alex Gordon-Weeks

**Affiliations:** 1 Department of General Surgery, Buckinghamshire Healthcare NHS Trust, London HP21 8AL, UK; 2 Bodleian Health Care Libraries, University of Oxford, Oxford OX3 9DU, UK; tatjana.petrinic@ouh.nhs.uk; 3 Department of Hepatobiliary and Pancreatic Surgery, Oxford University Hospitals NHS Foundation Trust, Oxford OX3 7LE, UK; michael.silva@ouh.nhs.uk (M.S.); zahir.soonawalla@ouh.nhs.uk (Z.S.); srikanth.reddy@ouh.nhs.uk (S.R.); 4 Nuffield Department of Surgical Sciences, University of Oxford, Oxford OX3 9DU, UK; alex.gordon-weeks@nds.ox.ac.uk

**Keywords:** pancreatic cancer, diagnosis, test, KRAS, mutation, mucus

## Abstract

This meta-analysis aims to identify the diagnostic accuracy of mutations in the Kirsten Rat Sarcoma (KRAS) oncogene in the diagnosis of pancreatic ductal adenocarcinoma (PDAC). The survival of PDAC remains poor often due to the fact that disease is advanced at diagnosis. We analysed 22 studies, with a total of 2156 patients, to identify if the detection of KRAS mutations from pancreatic exocrine secretions yields sufficient specificity and sensitivity to detect patients with PDAC amongst healthy individuals. The majority of the studies were retrospective, samples were obtained endoscopically or surgically, and included comparator populations of patients with chronic pancreatitis and pre-malignant pancreatic lesions (PanIN) as well as healthy controls. We performed several analyses to identify the diagnostic accuracy for PDAC among these patient populations. Our results highlighted that the diagnostic accuracy of KRAS mutation for PDAC was of variable sensitivity and specificity when compared with PanINs and chronic pancreatitis, but had a higher specificity among healthy individuals. The sensitivity of this test must be improved to prevent missing early PDAC or PanINs. This could be achieved with rigorous prospective cohort studies, in which high-risk patients with normal cross-sectional imaging undergo surveillance following KRAS mutation testing.

## 1. Introduction

Pancreatic ductal adenocarcinoma (PDAC) has a poor prognosis. Despite the enhanced understanding of PDAC biology [1], developments in pre-operative imaging [2], adjuvant/neoadjuvant chemotherapy [3,4], and image-guided surgery [5], there have been only marginal improvements in survival over the last two decades [6,7]. Resection of PDAC with clear surgical margins (R0) [8,9] and negative lymph nodes [10,11] predicts survivorship, indicating that early disease detection could improve outcomes. Currently, a significant proportion of patients present with advanced disease and so the development of diagnostic tests to identify pre-malignant or early-stage PDAC is vital. 

PDAC develops through the step-wise accumulation of mutations in tumour suppressor and oncogenes [12] accompanied by histological progression from benign precursor lesions (PanIN) to invasive malignancy and metastases [13,14]. Mutations in the Kirsten Rat Sarcoma (KRAS) oncogene are present in over 90% of resected PDAC specimens [15,16,17]. Most of these mutations are in KRAS codon 12, with rare mutations in codons 13 and 61 [15]. KRAS mutation is one of the earliest genetic events in PDAC evolution [15], being present in PanIN lesions prior to the development of invasive disease. Distant, incurable metastases occur much later; between 10–15 years following the acquisition of the initial KRAS mutation [16], indicating that there is a long period between the development of PanIN lesions which are not visible using cross-sectional imaging and incurable disease. Identification of KRAS mutation in at-risk individuals could therefore serve as a useful screening tool for the detection of curative disease, or pre-invasive lesions prior to appearance using conventional imaging modalities.

The past decade has seen burgeoning interest in the detection of cell-free DNA (cfDNA) for early cancer diagnosis [18,19,20,21,22]. Routinely used techniques to detect KRAS mutation include restriction-fragment length polymorphism (RFLP) analysis [17,18,19], qPCR-based techniques [20] or next-generation sequencing [21]. These techniques are highly sensitive and able to detect mutant KRAS present in 1% of cells within PDAC tissue [17,20]. However, the detection of circulating KRAS-mutant cfDNA has a sensitivity for the diagnosis of PDAC of only 20–25% [21,22]. This may result from the relatively low mutant allele fraction seen in PDAC [23], or because a PDAC needs to be relatively advanced before sufficient mutant DNA is released into the circulation. Sampling of pancreatic exocrine secretions may offer higher sensitivity because PDAC originates from the ductal epithelium and therefore mutant KRAS might be expected to be present in pancreatic secretions earlier in PDAC evolution than the point at which it enters the blood. The specificity of KRAS mutant cfDNA detection in pancreatic exocrine secretions may also be higher than blood, as mutation detected in the blood may develop from cancers at sites other than the pancreas. These include cancers of the lung or colon which frequently display KRAS mutation [24,25].

Here, we performed a meta-analysis of diagnostic accuracy studies for the detection of KRAS mutation in pancreatic exocrine secretions from PDAC patients. We were primarily interested in whether this approach yields adequate sensitivity and specificity to identify patients with PDAC amongst a healthy population. If KRAS mutation is detectable in the pancreatic secretions of PDAC patients but not healthy subjects at an acceptable sensitivity/specificity, this would provide an argument for prospective, population-based or randomised analysis of its use as a screening test in patients at high risk of PDAC based on family history or the presence of specific germ-line mutations.

## 2. Results

### 2.1. Study Inclusion

**Figure 1:**
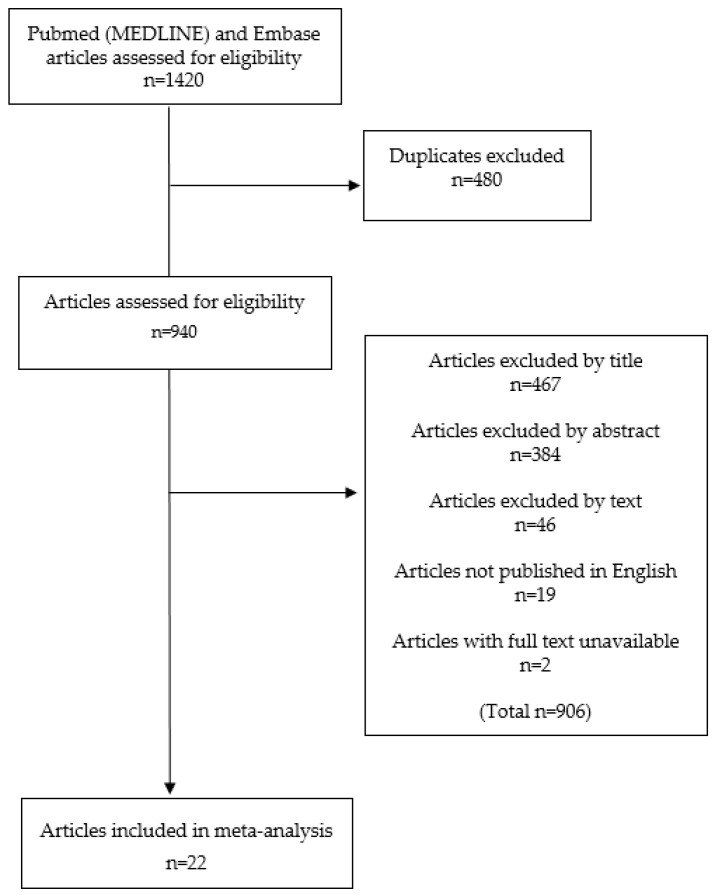
PRISMA flow diagram.

The included studies can be seen in the PRISMA flow diagram (Figure 1). We identified 22 studies inclusive of 2156 patients eligible for systematic review and meta-analysis after review of the full-text literature. No studies were identified from a search of the bibliographies of included studies. We made no attempt to contact authors of the included manuscripts as all outcome data were available within the publications meeting the inclusion criteria. The studies were generally of a moderate risk of bias (Table 1), with particularly poor use of follow-up to assess whether patients with a control group who are KRAS positive ultimately develop PDAC.

### 2.2. Study Demographics

The demographic details of the included studies are shown (Table 2). Studies were predominantly retrospective cohort studies, where the diagnosis of pancreatic cancer was known in a total of 850 patients at the time of mutant KRAS testing, equating to a pancreatic cancer prevalence of 39%. Comparative diagnoses of the study populations included pancreatitis, benign pancreatic cystic lesions or healthy patients. There were very few studies that included both groups of healthy controls and patients with pre-malignant lesions such that understanding the ability of KRAS mucus testing to differentiate between these two groups was not possible. Furthermore, although several studies included groups of patients with benign lesions, the histological subgroups (IPMN, mucinous cystic neoplasm, pseudopapillary neoplasm, etc.) were poorly documented.

We were unable to identify any prospective cohort studies which would be considered the gold-standard method for investigating the diagnostic accuracy of a test such as KRAS mutant testing. Studies that included healthy controls did not report the indication for endoscopic examination. Only four studies performed a median follow-up of over 5 years in order to determine whether patients with benign tumours or pancreatitis and KRAS mutation ultimately developed PDAC [29,33,38,40]

### 2.3. Mutant KRAS Determination.

All studies provided a good description of the methodology through which mucus, secretions and juice were obtained and analysed (Table 3). Most studies obtained pancreatic secretions through ERCP with some sampling from the duodenum and two studies obtaining secretions directly from the pancreatic duct at pancreatectomy. Approximately half of the studies used secretin stimulation to increase mucus yield. Reporting of the volume of mucus obtained was poor and the use of secretin stimulation did not appear to dramatically increase mucus yield. Included studies used similar DNA preparation techniques, with most utilising a phenol/chloroform-based extraction method followed by PCR-based amplification.

KRAS mutation was predominantly detected using the restriction fragment length polymorphism (RFLP) method with bands identified using gel electrophoresis. Some studies reported sequencing of the DNA fragments. More recent publications used alternative techniques including high-resolution melt analysis (HRMA) or real-time PCR-based methods (e.g., QuARTS) [46,48,49]. There were insufficient numbers of such studies to enable the comparison of sensitivity/specificity between techniques. Two studies used allele-specific amplification to selectively amplify the mutant allele. In most studies, pancreatic cancer was confirmed through subsequent histological analysis and this served as the gold-standard test on which mutation testing was benchmarked.

### 2.4. Assessment of Diagnostic Accuracy.

**Figure 2:**
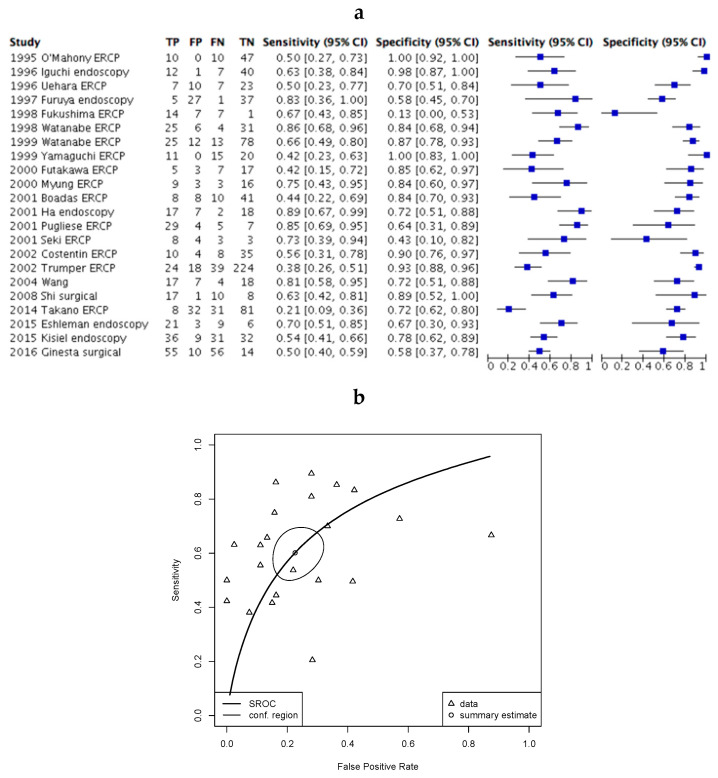
Kirsten Rat Sarcoma (KRAS) mutation testing in all patients across included studies. (**a**) Forest plot of included studies where error bars indicate the 95% confidence interval. True positive (TP), False positive (FP), False negative (FN), True negative (TN); (**b**) SROC plot of data demonstrating 95% confidence (conf.) region for the sensitivity/specificity estimate. The Partial area under the curve (restricted to observed estimates) is 0.71.

The diagnostic accuracy (sensitivity and specificity) of KRAS mutation testing was assessed across all included studies in an analysis that compared patients with pancreatic cancer with all other test subjects (pancreatitis, benign tumours and healthy patients). In this study, we have defined sensitivity as the proportion of patients with PDAC that tested positive for KRAS mutation, and specificity as the proportion of patients without PDAC that tested negative for KRAS mutation. Sensitivity (38%–89%) and specificity (13%–100%) varied widely across studies (Figure 2a). A summary receiver operating characteristic (ROC) curve constructed from this analysis confirmed significant heterogeneity in the diagnostic accuracy elicited across studies (Figure 2b).

**Figure 3:**
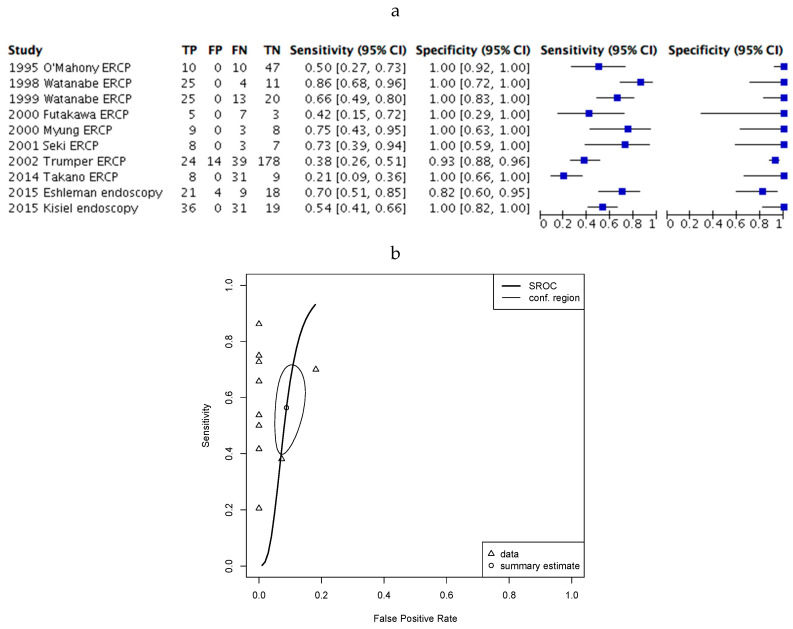
KRAS mutation testing in PDAC patients and healthy controls. (**a**) Forest plot of included studies where error bars indicate the 95% confidence interval; (**b**) SROC plot of data demonstrating 95% confidence (conf.) region for the sensitivity/specificity estimate. The Partial area under the curve (restricted to observed estimates) is 0.53.

We next questioned whether detection of KRAS mutation may be beneficial in the diagnosis of PDAC within a healthy population. This would be useful for high-risk patients with a family history of pancreatic cancer or patients carrying specific mutations relevant to PDAC risk but without imaging abnormality [50]. To do this we repeated the meta-analysis for healthy controls, excluding patients with a diagnosis of pancreatic cancer or benign pancreatic tumours. In this analysis (Figure 3a) the sensitivity was still highly variable (21%–86%), but specificity (82%–100%) improved significantly, with a reduction in heterogeneity. This was confirmed by analysis of the ROC curve (Figure 3b).

**Figure 4:**
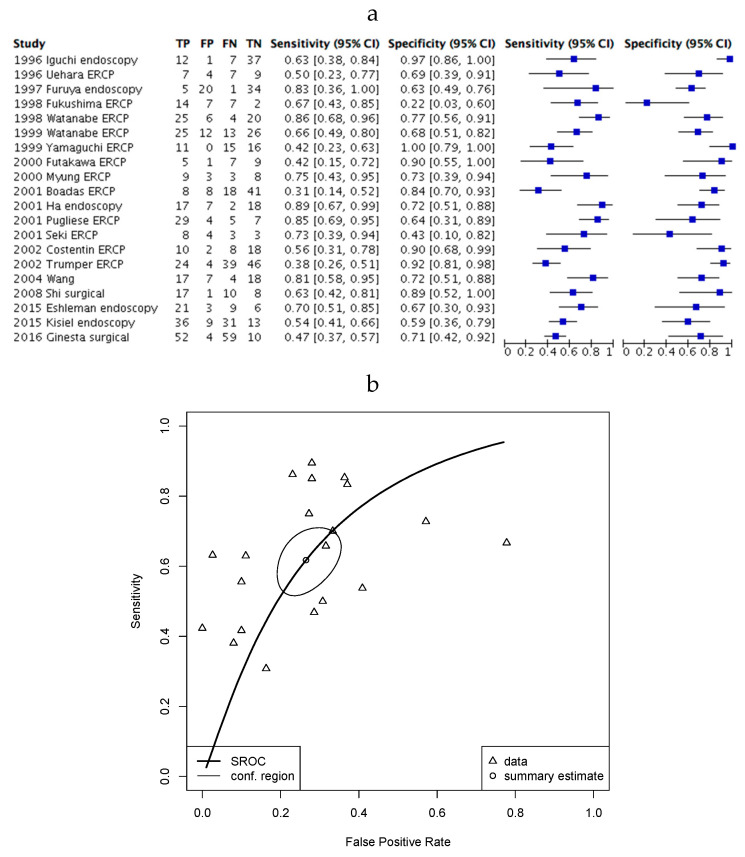
KRAS mutation testing in PDAC patients and patients with pancreatitis. (**a**) Forest plot of included studies where error bars indicate the 95% confidence interval; (**b**) SROC plot of data from demonstrating 95% confidence (conf.) region for the sensitivity/specificity estimate. The Partial area under the curve (restricted to observed estimates) is 0.67.

Finally, we analysed the diagnostic accuracy of KRAS mutation testing in a population of patients with pancreatitis (Figure 4). Here, the sensitivity (42%–89%) and specificity (22%–100%) was, again, highly heterogenous.

## 3. Discussion

In this study, we assessed the utility of detecting KRAS mutation in pancreatic secretions for the diagnosis of PDAC. If this technique can detect PDAC with adequate sensitivity and specificity then it may be applicable to screen high-risk individuals.

We identified a large number of studies that investigated KRAS mutation testing in the pancreatic secretions of PDAC patients, healthy controls and those with benign pancreatic pathology. First, it is clear that mutant KRAS is detectable in secretions from either the duodenum via standard endoscopy or the pancreaticobiliary system via ERCP. We did not identify sufficient studies comparing these approaches to enable understanding as to which provides optimal test accuracy. Studies using endoscopic methods to obtain samples used a single endoscopic modality alone; if sufficient samples could not be obtained via the single modality for a patient, that particular patient was excluded from the study. KRAS mutation was detected in PDAC patients, but also in a significant proportion of patients with pancreatitis or benign pancreatic tumours across multiple studies using a range of biochemical methodologies. Our findings show that KRAS mutation testing demonstrates a high degree of specificity once patients with pancreatitis or benign pancreatic tumours are excluded. This would, therefore, be applicable to a patient population at high risk of PDAC but with normal cross-sectional imaging. Several studies demonstrated that a high level of specificity was possible without appreciable loss of sensitivity [31,32,39,45]. High specificity is a useful feature of a screening test, as it ensures few patients receive unnecessary subsequent investigation or treatment that risk morbidity, or in the case of pancreatic resection, potential mortality.

Despite demonstrating promising specificity for the detection of PDAC in an otherwise healthy population, the sensitivity varied dramatically between studies, and in some, was too low to be of clinical use [34,42,44,46]. Heterogeneity of sensitivity estimates is an important finding and is likely multifactorial. Potential causes include variation in population demographics, differences in methods for exocrine secretion sampling or KRAS mutation detection and introduction of bias. We felt that the pooling of diagnostic accuracy estimates would have been misleading and methodologically unsound given the heterogeneity in study methodology and variability in diagnostic accuracy estimates between studies. Instead, we chose to display estimates as HSROC curves to aid understanding of estimate heterogeneity and identify potential links between this and study methodology.

Importantly, the study demonstrating the lowest sensitivity utilised DNA sequencing technology to detect KRAS mutation [42] and it is well recognised that this technique has a lower sensitivity than methods such as RFLP [47]. A second study demonstrating low sensitivity was that of Trumper et al. [42]; one of the few prospective studies identified. In this well-conducted study, the researchers went to lengths to reduce the false-positive rate and collected pancreatic juice following the injection of contrast into the biliary tree which diluted pancreatic secretions; both factors would have reduced test sensitivity. Finally, in the study by Kiesel et al. [46], which demonstrated high specificity but a sensitivity of only 54%, a real-time PCR-based mutation detection assay (QuARTS) was utilised. The sensitivity for this method is dependent upon a number of factors including PCR primer design, thermocycler settings, reagent chemistry and cut-off threshold. Any one or more of these factors may have contributed to the relatively low sensitivity demonstrated in this study.

Poor reporting, retrospective data collection, non-consecutive recruitment and analysis of a non-clinically relevant population with inflated disease prevalence all significantly over-estimate diagnostic test accuracy [50,51]. Several of the studies demonstrating the highest sensitivity fit several of these characteristics [31,35,39] and so are likely to have over-estimated test accuracy. Indeed, all included studies had a significantly higher disease prevalence (Table 2) than the general population, where approximately only four to six persons per 100,000 of the population will develop PDAC [52]. Therefore, its diagnostic performance in a population of high-risk individuals may be significantly lower than indicated here and this is obviously the predominant weakness of our approach. Further investigation in high-risk populations is therefore required.

Before being considered of clinical use for the screening of high-risk patients, test sensitivity would need to be improved to prevent false reassurance and miss underlying early PDAC or pre-malignant PanIN lesions. Sensitivity could be improved through a number of methods including repeated testing, improvements in pancreatic secretion capture and technological innovations that enable detection of mutation in lesser amounts of DNA. Because of the high rate of KRAS mutation in PDAC tumours and pre-neoplastic lesions, a combination of the perfect sampling method and mutation detection technology should allow for a sensitivity approaching 90% for discrimination between PDAC and the healthy state. Pancreatic secretion sampling is better suited to smaller, early pancreatic tumours or PanIN lesions, as duct obstruction prevents washout of pancreatic mucous and a reduction in the sensitivity with which mutant cfDNA is detected [34]. For the studies included here, there was relatively poor reporting of the PDAC disease stage, degree of ductal obstruction or anatomical location of the tumour within the pancreas and so it is difficult to understand the degree to which this affected sensitivity estimates.

Alternative methods for pancreatic secretion collection that may improve sensitivity include stool sampling, which has the benefit of remote sampling and is of a less invasive nature than endoscopic sampling. This method has a reported sensitivity of up to 82% for the detection of KRAS mutation in PDAC patients [53,54], however, it is likely to have a lower specificity given the propensity for mucus to be generated by benign or malignant colorectal polyps which also frequently harbour KRAS mutation [24]. The development of more advanced KRAS mutant detection methods will also improve test sensitivity [55]. This is the case for droplet digital PCR [56], a method ideally suited to the detection of mutations existing in samples such as pancreatic secretions which contain low levels of nucleic acid and suffer from abundant protein contamination. Alternatively, incorporation of multiple genetic indicators such as panels of mutations, DNA methylation status [46,54] or simultaneous tumour marker assessment [55] could be used to improve sensitivity.

Finally, we were able to assess the role of mutant KRAS testing for distinguishing between PDAC and benign or pre-malignant pancreatic tumours. Our data indicate that the test specificity is too low to be used alone for this purpose; a finding supported by analyses of tissue from benign pancreatic lesions, which frequently display KRAS mutation [44]. It is possible that patients with benign tumours and KRAS mutation from pancreatic exocrine secretions go on to develop PDAC, however, there was insufficient long-term follow-up in the included studies to enable this to be investigated.

Analysis of pancreatic exocrine secretions may be improved by the inclusion of other mutations known to be associated with particular tumour subtypes including GNAS mutations in intraductal papillary neoplasms of the bile ducts (IPNBs), or von Hippel -Lindau (VHL) mutation in serous cystic neoplasms [44,57,58,59]. Equally, in patients with a visible cyst on cross-sectional imaging, the use of endoscopic ultrasound and cyst aspiration as a means of sampling cfDNA is likely to be the best approach for differentiating between pre-malignant tumour subtypes [58,59,60]. Here, we chose to exclude studies utilising Endoscopic Ultrasound (EUS) as the technical expertise required to perform this investigation are unlikely to be available at the volume required for a screen-based investigative strategy and our primary interest was to determine whether KRAS mutation is detectable through simple endoscopic means. Furthermore, in the high-risk populations, because diabetes or pancreatitis is more likely rather than the presence of a cystic neoplasm on cross-sectional imaging due to family history, EUS is less likely to be diagnostically useful.

The limitations of our meta-analysis arise from the overall moderate risk of bias of the studies included, as well as the high level of heterogeneity between the studies which limits the amount of between-study comparisons that can be made. Furthermore, most studies were low power, retrospective in data collection and non-consecutive in recruitment which limits the conclusions that can be drawn. The analysis of publication bias is an important component of meta-analysis, however, robust methods for its analysis in diagnostic accuracy studies have not yet been developed, and as such, we were unable to assess for this [61]. It should also be recognised that there will be variation in the sensitivity/specificity of the test within each included study, resulting from demographic variation between subjects, disease stage, variability in the way the test is performed and the specific definition of a true positive test. Because individual patient-level data were not accessible, an understanding of this variation could not be reached in our analysis. A total of 19 articles were excluded because they were not published in English; these may have been high-quality studies that could have yielded interesting findings to contribute towards our meta-analysis. However, highly accurate translations would be required to ensure the studies can be reliably incorporated into our meta-analysis. Additionally, two studies were excluded because the full-text articles were not available. Despite our attempts at contacting the authors, we were not able to access these articles and therefore were unable to include potentially useful data into our study.

## 4. Materials and Methods

The meta-analysis was conducted according to the Meta-analysis of Observational studies in Epidemiology (MOOSE) group and Preferred Reporting Items for Systematic Reviews and Meta-analysis (PRISMA) protocols in diagnostic test accuracy [62,63].

### 4.1. Search Strategy

A structured literature search of MEDLINE (PubMed) and Embase databases was performed on Ovid by a health care librarian (T.P) on 13th November 2018. The search string included the following terms, “genes,” “ras,” “codon,” “KRAS,” “K RAS,” “Ki RAS,” “codon 12,” “codon 13,” “pancreatic neoplasm”, “adenocarcinoma”, “common bile duct neoplasms,” “biliary tract neoplasm”, “pancreatitis”, “intestinal secretions”, “fluid OR juice OR secretion,” “mucus OR mucosa”, “aspirate”, “stricture”, “mutation OR mutant OR alter OR variant OR variation OR modification.” Boolean operators and MeSH (Medical Subject Headings) terms were exploded to combine these keywords.

All conference abstracts were excluded which was specified in the search string. These searches yielded 708 and 712 articles from MEDLINE (PubMed) and Embase respectively, giving a total of 1420 articles.

Firstly, all duplicate articles and those not published in English were excluded. Following this, articles were systematically excluded by title and then abstract. The full text of the resulting articles were reviewed to identify those that met inclusion and exclusion criteria. Additional studies were identified from searching the reference lists of these articles.

### 4.2. Inclusion/Exclusion Criteria

Studies in humans were included where samples for identification of KRAS mutations were taken from duodenal or pancreatic secretions. Articles were included if ductal fluid was aspirated during endoscopic retrograde cholangiopancreatography (ERCP) or endoscopy, or if obtained from surgically resected specimens via pancreatoduodenectomy. However, studies with samples obtained from ERCP brushings or biopsy, Endoscopic Ultrasound with Fine Needle Aspirate (EUS-FNA) samples, or endoscopic aspirate of duodenal fluid through a cap over the major duodenal papilla were excluded. Furthermore, review articles were excluded as were articles where samples were obtained from circulating free or cellular DNA alone. Articles including patients with benign pathology including intraductal papillary mucinous neoplasm (IPMN), chronic pancreatitis, mucinous cystic neoplasm (MCN) and other benign pancreatic cysts were only included if the study also included patients with PDAC. Animal studies, case series with fewer than 10 patients and articles on basic science without human subjects were all excluded. Finally, articles not published in English, and studies where full-text was unavailable were also excluded.

### 4.3. Data Extraction and Statistics

Data were extracted by two authors (N.P. and A.G-W.) with discussion and review performed for any discrepant data. Risk of bias for each study was assessed by one author (N.P.), using a “Quality of Assessment of Diagnostic Accuracy Studies-2” (QUADAS-2) tool (University of Bristol, Department of Population Health Sciences, Bristol, UK) in this meta-analysis.

Study demographics and parameters related to sampling from specimens were extracted from studies and tabulated. Forest plots were generated using Review Manager version 5.3 (Cochrane, UK) and SROC curves for comparing KRAS detection in patients with pancreatic cancer with healthy individuals, those with chronic pancreatitis, and benign pancreatic pathology were generated using R. A bivariate random-effects model was used to generate 95% confidence regions using the media package in R [64].

## 5. Conclusions

In summary, this meta-analysis indicates that the sensitivity of KRAS mutation testing in endoscopically sampled pancreatic mucus varies considerably and is not currently ready to be used as a diagnostic biomarker. This study identified that positive KRAS mutation has utility in discriminating PDAC from healthy controls due to its high specificity. There remains significant scope for future work in this field and the focus should be on performing rigorous prospective cohort studies, in which high-risk patients with normal cross-sectional imaging undergo surveillance following KRAS mutation testing.

## Figures and Tables

**Table 1:** Quality of Assessment of Diagnostic Accuracy Studies-2 (QUADAS-2) table for quality assessment of studies of diagnostic accuracy.

Study	Risk of Bias				Concerns Regarding Applicability		
	Patient Selection	Index Tests	Reference Standard	Flow and Timing	Patient Selection	Index Tests	Reference Standard
O’Mahony [26]	Low	Low	Unclear	Low	Low	High	Low
Iguchi [27]	Unclear	Low	Low	High	Low	Low	Low
Uehara [28]	Low	Unclear	High	High	Low	Unclear	Low
Furuya [29]	Low	Low	Low	Unclear	Low	Unclear	Low
Fukushima [30]	High	High	Low	Low	Low	High	Low
Watanabe (1998) [31]	Low	Unclear	Low	Unclear	Low	Unclear	Unclear
Watanabe (1999) [32]	Low	Unclear	High	Low	Low	Low	Low
Yamaguchi [33]	High	Low	Low	Unclear	Low	Unclear	Low
Futakawa [34]	Low	High	High	Unclear	Low	Low	Low
Myung [35]	Low	High	Unclear	Low	Low	High	Unclear
Boadas [36]	High	Unclear	Unclear	High	Low	Unclear	Unclear
Ha [37]	High	Unclear	Low	High	Low	Low	High
Pugliese [38]	High	High	High	High	Low	High	Unclear
Seki [39]	Unclear	Unclear	Unclear	Unclear	Low	Unclear	Low
Costentin [40]	High	High	Unclear	High	Low	Low	Unclear
Wang [41]	High	Low	High	High	Low	Unclear	Low
Trumper [42]	Unclear	Low	High	High	Low	Low	Low
Shi [43]	Unclear	Low	Low	Unclear	Low	Low	Low
Takano [44]	High	High	Unclear	Low	Low	Low	Low
Eshleman [45]	High	Unclear	Low	Low	Low	Unclear	Low
Kisiel [46]	High	Low	Low	Unclear	Low	Low	Low
Ginesta [47]	High	Low	Unclear	Unclear	Low	Low	Low

**Table 2:** Study demographics.

Author	Published	Country	Design	Number of Patients (% PDAC Prevalence)	Patient Population	Age	Male (%)
O’Mahony [26]	1995	UK	*	67 (30)	PDAC, H	*	*
Iguchi [27]	1996	Japan	*	60 (32)	PDAC, Pc, B	*	*
Uehara [28]	1996	Japan	Ret-Co	47 (30)	PDAC, Pc, B, H	*	*
Furuya [29]	1997	Japan	Pro-Co	70 (8.6)	PDAC, Pc, B	*	*
Fukushima [30]	1998	Japan	Ret-Co	29 (72)	PDAC, Pc	*	*
Watanabe (1998) [31]	1998	Japan	Ret-Co	66 (44)	PDAC, Pc, H	* (39–83)	55
Watanabe (1999) [32]	1999	Japan	Ret-Co	140 (43)	PDAC, Pc, B, H	61 (28–84) ^§^	*
Yamaguchi [33]	1999	Japan	Ret-Co	46(57)	PDAC, Pc, B	*	*
Futakawa [34]	2000	Japan	Pro-Co	52 (23)	PDAC, Pc, B, H	*	*
Myung [35]	2000	Korea	Ret-Co	31 (39)	PDAC, Pc, H	63 (46–77) ^±^	61
Boadas [36]	2001	Spain	Pro-Co	90 (20)	PDAC, Pc	*	*
Ha [37]	2001	Japan	Ret-Co	44 (43)	PDAC, Pc	* (17–81)	*
Pugliese [38]	2001	Italy	Pro-Co	45 (76)	PDAC, Pc	66 (44–88) ^§^	56
Seki [39]	2001	Japan	Ret-Co	36 (47)	PDAC, Pc, H	*	72
Costentin [40]	2002	France	Ret-Co	57 (32)	PDAC, Pc, B	*	*
Wang [41]	2002	Germany	Pro-Co	358 (33)	PDAC, Pc, B, H	*	*
Trumper [42]	2004	Japan	Ret-Co	46 (46)	PDAC, Pc	*	*
Shi [43]	2008	USA	Ret-Co	36 (75)	PDAC, Pc	*	*
Takano [44]	2014	Japan	Ret-Co	152(26)	PDAC, Pc, B, H	65 (35–85) ^§^	58
Eshleman [45]	2015	USA	Cas-Co	272 (11)	PDAC, B, H	57^±^	48
Kisiel [46]	2015	USA	Cas-Co	102 (60)	PDAC, Pc, H	64 (49–76) ^§^	52
Ginesta [47]	2016	Spain	Cas-Co	135 (82)	PDAC, Pc, B	68 (40–79) ^±^	60

Pancreatic adenocarcinoma (PDAC), pancreatitis (Pc), Benign cystic lesion (B), healthy control (H), Retrospective cohort (Ret-Co), Prospective cohort (Pro-Co), Case-control (Cas-Co), ^§^ median (range), ^±^ mean (range), * missing data.

**Table 3:** Test characteristics.

Author	Modality	Site	Secretin Stimulation	Mucus Volume (mls)	DNA Extraction Method	Amplification Method	Mut-KRAS Detection Method	Codon/Mutant Tested	Gold Standard Test
O’Mahony [26]	ERCP	Bile duct	n	*	Pheno-chlor	PCR	RFLP	12	Histology
Iguchi [27]	Endoscopy	Duodenum	y	(30–40)	Pheno-chlor	PCR	RFLP, sequencing	12	Histology
Uehara [28]	ERCP	Pancreas	n	*	*	PCR	Slot-blot	12	*
Furuya [29]	Endoscopy	Duodenum	Y	0.5 ^±^	Acet-chlor	PCR	RFLP	12	Histology
Fukushima [30]	ERCP	Pancreas, bile duct	n	*	Pheno-chlor	PCR	RFLP	12	Histology
Watanabe (1998) [31]	ERCP	Pancreas	n	*	*	PCR	Hybridization probe	12	Histology
Watanabe (1999) [32]	ERCP	Pancreas	n	(2–3)	Pheno-chlor, Prot-K	PCR with A-sA		12	Histology
Yamaguchi [33]	ERCP	Pancreas	y	*	Pheno-chlor	PCR	SSCP	12	Histology
Futakawa [34]	ERCP	Pancreas	n	0.1	Pheno-chlor	PCR	RFLP	12	*
Myung [35]	ERCP	Pancreas	y	0.1	*	PCR	RFLP	12	Histology
Boadas [36]	ERCP	Pancreas	y	4.6 ^±^	*	PCR	RFLP	12	Histology
Ha [37]	Endoscopy	Duodenum	y	*	Pheno-chlor	PCR with A-sA	RFLP	12	Histology
Pugliese [38]	ERCP	Pancreas	n	*	*	PCR	RFLP, sequencing	12	Histology
Seki [39]	ERCP	Pancreas	y	2 ^±^	*	PCR	SSCP	12	*
Costentin [40]	ERCP	Pancreas	n	*	*	PCR	RFLP	12	*
Wang [41]	ERCP	Pancreas	n	(1–2)	*	RFLP-Targeted enrichment	RFLP, sequencing	12	*
Trumper [42]	Endoscopy	Duodenum	y	(10–15)	Pheno-chlor	PCR with A-sA	RFLP	12	Histology
Shi [43]	Surgery	Pancreas	n	*	*	PCR	qPCR-primer based	G12V, G12D, G12R	Histology
Takano [44]	ERCP	Pancreas	n	*	Proprietary (QiAMP^®^Kit)	PCR	DNA sequencing	G12D, G12R, G12V, Q61H	Histology
Eshleman [45]	Endoscopy	Pancreas	y	(5–10)	*	*	HRMA		*
Kisiel [46]	Endoscopy	Duodenum	y	2 ^±^	*	*	QuARTS	G12D	*
Ginesta [47]	Surgery	Pancreas	n	*	Pheno-chlor	PCR	qPCR primer-based	G12C, G12V, G12D, G12A, G12s, G12R, G13D	Histology

* missing data, ^±^ mean, phenol chloroform (Pheno-chlor), acetate-chloroform (Acet-chlor), proteinase-K (Prot-K), Single-stranded conformation polymorphism (SSCP), High-resolution melt analysis (HRMA), Quantitative allele-specific real-time target and signal amplification (QuARTS), Allelic-specific amplification (A-sA).
